# Role of CD40 ligation in dendritic cell semimaturation

**DOI:** 10.1186/1471-2172-13-22

**Published:** 2012-04-26

**Authors:** Anna-Maria Gerlach, Alexander Steimle, Lea Krampen, Alexandra Wittmann, Kerstin Gronbach, Julia Geisel, Ingo B Autenrieth, Julia-Stefanie Frick

**Affiliations:** 1 Institute for Medical Microbiology and Hygiene, University Hospital of Tübingen, 72076 Elfriede-Aulhorn-Str. 6, Tübingen, D-72076, Germany

**Keywords:** Dendritic cells, CD40 ligation, Maturation, Cytokine, MAP Kinase, Homoeostasis, T-cell

## Abstract

**Background:**

DC are among the first antigen presenting cells encountering bacteria at mucosal surfaces, and play an important role in maintenance of regular homeostasis in the intestine. Upon stimulation DC undergo activation and maturation and as initiators of T cell responses they have the capacity to stimulate naïve T cells. However, stimulation of naïve murine DC with *B. vulgatus* or LPS at low concentration drives DC to a semimature (sm) state with low surface expression of activation-markers and a reduced capacity to activate T-cells. Additionally, semimature DC are nonresponsive to subsequent TLR stimulation in terms of maturation, TNF-α but not IL-6 production. Ligation of CD40 is an important mechanism in enhancing DC maturation, function and capacity to activate T-cells. We investigated whether the DC semimaturation can be overcome by CD40 ligation.

**Results:**

Upon CD40 ligation smDC secreted IL-12p40 but not the bioactive heterodimer IL-12p70. Additionally, CD40 ligation of smDC resulted in an increased production of IL-6 but not in an increased expression of CD40. Analysis of the phosphorylation pattern of MAP kinases showed that in smDC the p38 phosphorylation induced by CD40 ligation is inhibited. In contrast, phosphorylation of ERK upon CD40 ligation was independent of the DC maturation state.

**Conclusion:**

Our data show that the semimature differentiation state of DC can not be overcome by CD40 ligation. We suggest that the inability of CD40 ligation in overcoming DC semimaturation might contribute to the tolerogenic phenotype of semimature DC and at least partially account for maintenance of intestinal immune homeostasis.

## Background

Dendritic cells (DC) are among the first antigen presenting cells encountering bacteria at mucosal surfaces and play an important role in maintenance of regular homeostasis in the intestine. Stimulation of DC with e.g. TLR agonists leads to activation and maturation of DC by activation of NF-κB and mitogen-activated protein kinase (MAPK) family members [1]. This results in a rapid production of costimulatory molecules, cytokines and pro-inflammatory mediators that affect T-cell differentiation, for instance.

We identified *Escherichia coli* mpk, a commensal *E. coli* strain which induces colitis in genetically predisposed hosts and *Bacteroides vulgatus* mpk which does not elicit colitis and even prevents the colitis caused by *E. coli* mpk [2,3]. Stimulation of bone marrow derived dendritic cells (BMDC) with *E. coli*[4] or lipopolysaccharide (LPS) at high concentration [5] induced TNF-α, IL-12 and IL-6 secretion and expression of activation-markers, whereas stimulation with *B. vulgatus* or [4] LPS at low concentrations [5] only led to secretion of IL-6 and DC were driven to a semimature state with low expression of activation-markers. Those semimature DC were nonresponsive to subsequent TLR stimulation in terms of maturation and TNF-α but not IL-6 production [4,5]. Moreover, the low positive expression of activation surface marker like e.g. CD40 on semimature DC, was not overcome by a subsequent stimulus via TLR4 [4]. This might contribute to the reduced activation of T-cells by semimature DC [4] as binding of the CD40 ligand (CD40L) on naïve T-cells to CD40 is a crucial signal for generation of effective CD4^+^ and CD8^+^ T-cell responses [6,7]. CD40 ligation results in upregulation of CD83, CD80 and CD86 as well as MHC molecules on DC. Additionally, the expression of adhesion molecules ICAM-1 and CD58 [8-11] is upregulated and survival of DC is supported by CD40 ligation [12,13]. Furthermore, CD40 ligation of mature DC results in secretion of proinflammatory cytokines e.g. IL-1, IL-6 and IL-12 [9-11][14,15].

IL-12 plays an important role in T-cell polarization by promoting Th-1 responses. Its bioactive heterodimer IL-12p70 consists of a p40 and p35 subunit, which are encoded by different genes and therefore independently regulated. IL-12p40 can also form homodimers (IL-12p80) which were shown to inhibit IL-12p70 mediated immune responses [16,17].

Mitogen-activated protein kinase (MAPK) signal transduction pathways play a crucial role in many aspects of immune mediated inflammatory responses [18]. The MAPK ERK, JNK and p38 are important regulators of host immune responses to e.g. bacterial stimuli. Extracellular stimuli induce phosphorylation of MAPK–kinase-kinase (MKKK) which in turn phosphorylate MKK. Specific MKK are necessary to phosphorylate and activate MAPK, which results in activation of downstream kinases and transcription factors [18-20]. The products of inflammatory genes include e.g. cytokines, chemokines and adhesion molecules which promote recruitment of immunocompetent cells to inflammatory sites. Additionally, the MAPK p38 enhances the mRNA stability of many proinflammatory cytokines, e.g. IL-8, TNF-α or IL-6 [21-23].

Within the present study we analyzed the effects of DC semimaturation on cellular responses to CD40 ligation and showed that the semimature differentiation state of DC, induced by stimulation with *B. vulgatus* or LPS^lo^ can not be overcome by CD40 ligation.

## Methods

### Mice

C57Bl/6x129sv mice were obtained from own breeding. All mice were kept under SPF conditions. Male and female mice were sacrificed at 6–12 weeks of age. Animal experiments were reviewed and approved by the responsible institutional review committee (Regierungspräsidium Tübingen, December 19^th^ 2008).

### Abs and reagents

Ultra pure LPS *Salmonella enterica* serovar Minnesota was purchased from Calbiochem (Schwalbach, Germany). The following antibodies were used for flow cytometry: PE conjugated anti-mouse CD11c, clone HL3, Biotin conjugated anti-mouse CD40, clone 3/23, Biotin conjugated anti-mouse CD80, clone 16-10A1, FITC conjugated anti-mouse I-A/I-E clone 2 G9, FITC conjugated anti-mouse CD86, clone GL1. As isotype control hamster IgG_1_ λ2, hamster IgG_2a_ κ and rat IgG_2a_ κ were used. For CD40 ligation we used purified NA/LE hamster anti-mouse CD40, clone HM40-3, and purified NA/LE hamster IgM, λ1 isotype control, clone G235-1 (all antibodies from BD, Heidelberg, Germany). p38 MAP kinase inhibitor (SB202190) was purchased from Calbiochem (Schwalbach, Germany), and ERK inhibitor (PD98059) from Promega (Mannheim, Germany).

### Western blotting

For p38 and pERK Western blot analysis proteins (50 μg) were solubilized in Laemmli sample buffer. They were separated on SDS-PAGE gels and transferred to nitrocellulose membranes. The membranes were blocked for 1 h at room temperature in 5% dry milk in TBS/T (TBS containing 2,01% Tween). After that the membranes were incubated with mouse anti-p38 MAPK (pT180/pY182) or with mouse anti-ERK 1/2 (pT202/pY204) (both BD Pharmigen, Heidelberg, Germany) at 4°C over night. The antibody solution was diluted 1:1000 in 5% dry milk in TBS/T. After incubation the membranes were washed three times in TBS/T and were treated with the secondary antibody (polyclonal rabbit anti-mouse conjugated to horseradish peroxidase, DakoCytomation, Hamburg, Germany; diluted 1:1000 in 5% dry milk in TBS/T) for 2.5 h at room temperature. After repeating the washing step the proteins were detected by enhanced chemiluminescence. Before using ß-actin (mouse anti-mouse β-actin; Sigma, Munich, Germany) as a control for protein loading, the blots were stripped for 20 min with 10 ml stripping-solution (10 mM NaOH and 250 mM guanidinium chloride).

### Bacteria and cell lines

The bacteria used for stimulation of the murine dendritic cells were *Escherichia coli* mpk [2] and *Bacteroides vulgatus* mpk [2] . The *E. coli* strain was grown in Luria-Bertani (LB) medium under aerobic conditions at 37°C. *Bacteroides vulgatus* was grown in Brain-Heart-Infusion (BHI) medium and anaerobic conditions at 37°C. In some experiments, J558L/CD40L cells were used for CD40 ligation. The cells were cultured in DMEM (*Dulbecco’s modified Eagle’s medium*,Invitrogen, Darmstadt, Germany) supplemented with 1 g/l glucose, L-glutamine, pyruvate, 50 μmol/l 2-mercaptoethanol, 10% FCS and penicillin/streptomycin.

### Mouse DC isolation

Bone marrow cells were isolated and cultured as described previously [4] with minor modifications. Cells were harvested at day 7 and used to evaluate the effects of cellular challenge with *E. coli* mpk, *B. vulgatus* mpk and LPS on subsequent CD40 ligation. Cytokine release and expression of surface markers were determined after CD40 ligation as described below.

### Stimulation of isolated DC

At day 7, DC were stimulated with viable bacteria at a MOI of 1 at 37°C, 5% CO_2_. Gentamicin was added one hour after stimulation and cells were incubated for 24 hour. To exclude bacterial overgrowth, CFU of viable bacteria was determined at the end of incubation period. Respectively, DC were stimulated with LPS (1 ng/ml and 1 μg/ml). After 24 h cell culture supernatant was harvested for analysis of cytokine expression and cells were used for flow cytometry of surface marker expression.

### CD40 ligation

To determine the effects of CD40 ligation on DC cytokine production and expression of surface markers DC were restimulated using an agonistic anti-CD40 mAb (BD, Heidelberg, Germany). Therefore, DCs pretreated with *E. coli, B. vulgatus* or LPS were harvested, washed twice and cultured at 1.5x10^5^ DC in the presence of 5 μg/ml anti-CD40 mAb in DC culture medium at 37°C, 5% CO_2_. As a control, DCs were incubated with 5 μg/ml of the IgM isotype control antibody (BD, Heidelberg, Germany). After 48 h, DC culture supernatants were harvested and analyzed for cytokine concentrations by ELISA. The expression of CD80 and CD86 on the DC surface was determined by FACS analysis.

For determination of CD40 expression of DC upon CD40 ligation the J558/LCD40L cell line was irradiated with 180 Gy in a Gammacell 1000 Elite® (Nordion, Ottawa, Canada) prior to co-culture with DC. 5x10^4^ J558L/CD40L cells were cultured with 1.5x10^5^ DC in DC culture medium for 48 h at 37°C, 5% CO_2._ DC were harvested and analyzed for expression of CD40 by FACS.

### Inhibition of MAP kinase signaling

DC were incubated with the p38 MAP kinase inhibitor SB202190 (2 μmol/l) or the ERK inhibitor PD98059 (50 μmol/l) for 30 min prior to CD40 ligation. After 30 min the cells were washed. CD40 ligation with anti CD40 mAb was performed for 24 h. Cell culture supernatants were harvested and used for determination of cytokine concentrations.

### Cytokine analysis by ELISA

For analysis of IL-6, IL-12p40 and IL-12p70 concentrations in cell culture supernatants commercially available ELISA kits (BD, Heidelberg, Germany) were used according to the manufacturer’s instructions.

### Flow cytometry analysis

3x10^5^ DC were incubated in 150 μl PBS containing 0.5-μl of fluorochrome conjugated antibodies and applied to flow cytometry analysis. 30,000 cells were analyzed using a FACS Calibur (BD, Heidelberg, Germany).

### Statistical analysis

Statistical analysis was performed using the two sided unpaired Student’s *t*-test. P values < 0.05 were considered significant. Error bars represent ± SEM.

## Results

### CD40 ligation does not overcome DC semimaturation

Stimulation of DC with *B. vulgatus* or LPS^lo^ (1 ng/ml) leads to induction of DC semimaturation [4,5] whereas stimulation of immature DC with *E. coli* or LPS^hi^ (1 μg/ml) induces DC maturation. The semimature DC phenotype is characterized by tolerance towards a subsequent stimulation via TLR2 or TLR4 in terms of TNF-α and IL-12p70 but not IL-6 secretion and a low positive expression of costimulatory molecules like e.g. CD40, CD80 CD86 [4,5]. Herein we investigated whether CD40 ligation as a TLR independent DC activation signal can overcome the semimature DC phenotype and induce activation and maturation of semimature DC.

**Figure 1:**
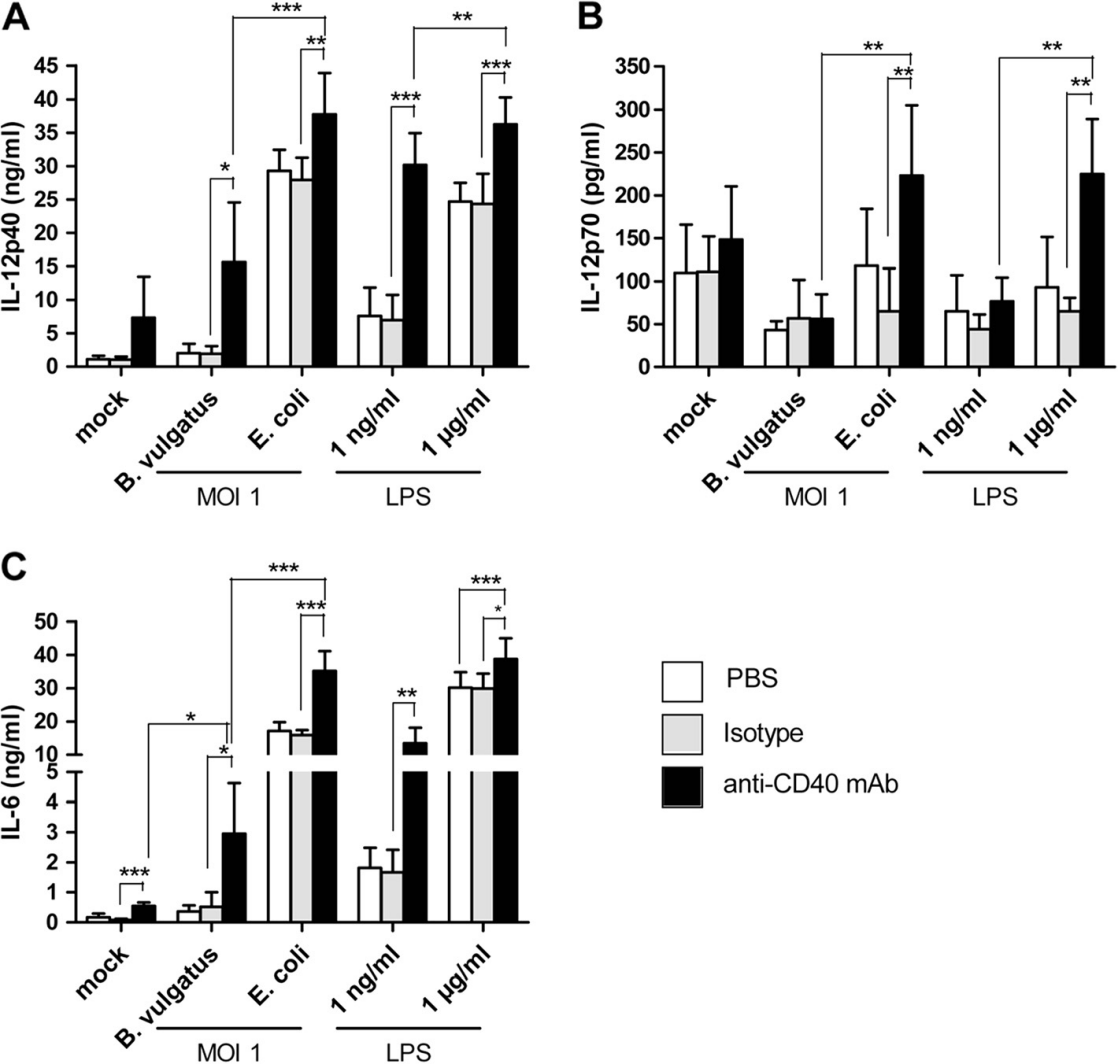
**Cytokine secretion of differentially primed BMDC in response to secondary CD40 ligation.** Wildtype BMDC were stimulated with B. vulgatus mpk (MOI 1) or E. coli mpk (MOI 1) and LPS at low (1 ng/ml) or high concentration (1 μg/ml), respectively, for 24 hour. Unstimulated DC (PBS) served as a control. Subsequently, these DC were washed twice and were re-stimulated with an agonistic anti-CD40 mAb (5 μg/ml) for 48 hours (black bars). As a control, DC were further incubated in medium only (white bars) or in the presence of the isotype control (grey bars). The concentrations of IL-12p40 (**A**), IL-12p70 (**B**) and IL-6 (**C**) were determined by ELISA. The bars represent the mean values of three independent experiments, each performed as duplicate, + SD. * p < 0.05, ** p < 0.01, *** p < 0.001.

By stimulation of immature DC with *B. vulgatus* or LPS^lo^ we induced semimature DC, and by stimulation with *E. coli* or LPS^hi^ DC maturation was induced. Secretion of IL-12p40 (Figure 1A), IL-12p70 (Figure 1B) and IL-6 (Figure 1C) was determined by ELISA. CD40 ligation of semimature DC led to secretion of IL-12p40 but not to secretion of the bioactive heterodimer IL-12p70. In contrast, CD40 ligation of mature DC resulted in significant enhanced secretion of IL-12p70 as compared to cells treated with the anti-CD40 mAb isotype control. Mature DC revealed a high spontaneous production of IL-12p40 which was only slightly enhanced by CD40 ligation (Figure 1A, B). Additionally, CD40 ligation of semimature DC resulted in increased levels of IL-6 compared to immature DC. In mature DC which also showed a high spontaneous secretion rate of IL-6 CD40 ligation only led to a slight further increase of IL-6 production (Figure 1C).

**Figure 2:**
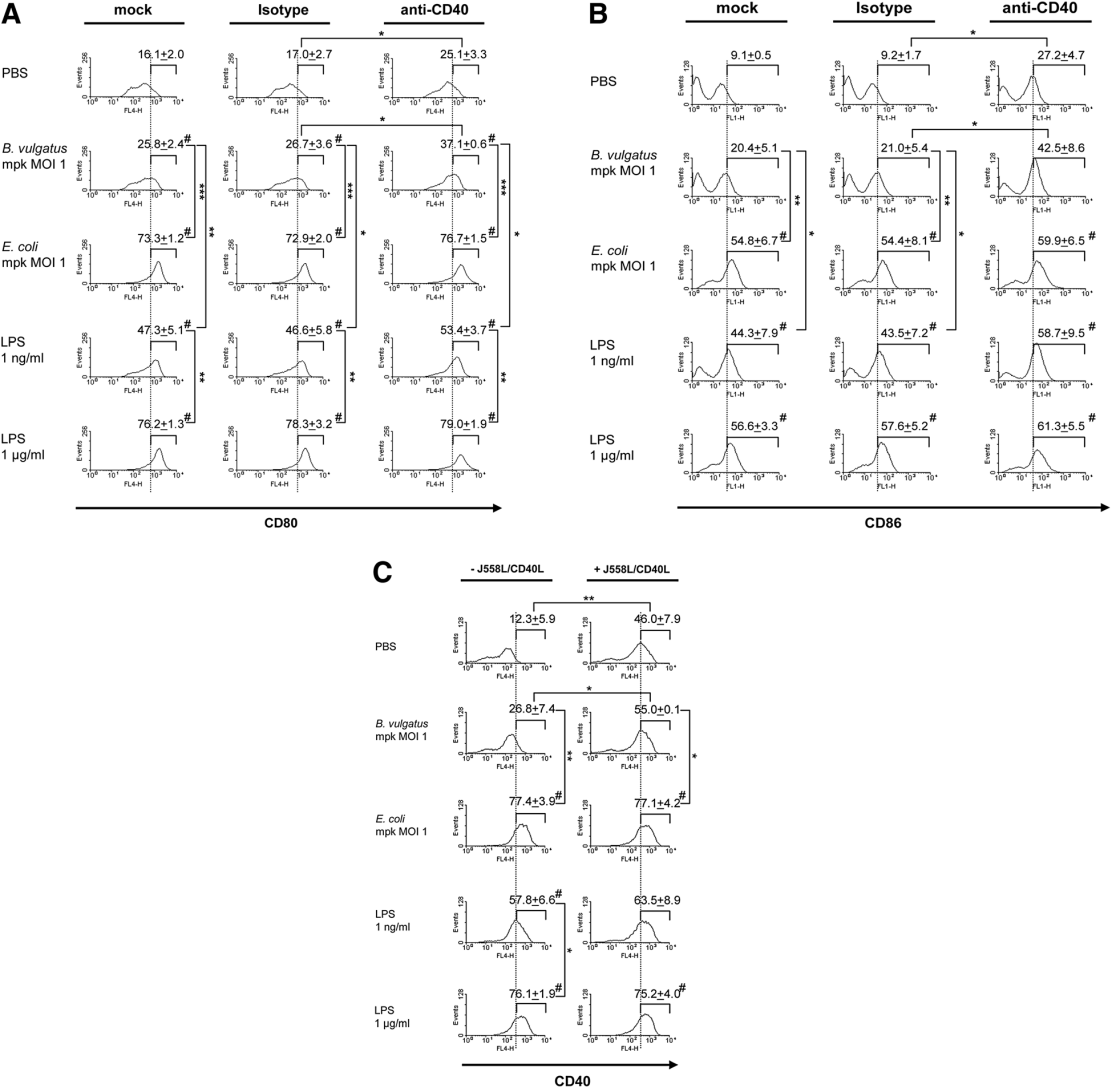
**Expression of CD80, CD86 and CD40 on differentially primed BMDC after secondary CD40 ligation.** Wildtype BMDC were stimulated with B. vulgatus mpk (MOI 1) or E. coli mpk (MOI 1) and LPS at low (1 ng/ml) or high concentration (1 μg/ml), respectively, for 24 hours. Unstimulated DCs (PBS) served as a control. **Figure**2**A** and 2**B**: The pre-treated DC were washed twice and were re-stimulated with an agonistic anti-CD40 mAb (5 μg/ml) for 48 hours. As a control, DCs were further incubated in medium only (mock) or in the presence of the isotype control. The expression levels of CD80 (**A**) and CD86 (**B**) were measured by FACS analysis. **Figure**2**C**: The pre-treated DC were washed twice and were re-stimulated with CD40L by co-culture with J558L/CD40L transfectants or were cultured in medium only as a control (− J558L/CD40L). After 48 hours, the expression of CD40 was determined by FACS analysis. Each histogram is representative of three independent experiments. The data are mean values of the percentages of the positive cell populations determined in these three experiments, ± SD. * p < 0.05, ** p < 0.01, *** p < 0.001; # p < 0.05 compared to the respective control (PBS).

Next, we investigated whether CD40 ligation can overcome the low positive expression of DC activation markers on semimature DC. Therefore, we analysed the expression of CD80 (Figure 2A), CD86 (Figure 2B), and CD40 (Figure 2C) on immature, mature and semimature DC upon CD40 ligation in comparison to mock cells or cells treated with the anti-CD40 mAb isotype control by FACS.

On semimature DC the expression of CD80 was slightly enhanced upon CD40 ligation, as compared to the control cells (mock or isotype treated). However, in comparison to mature DC, in semimature DC the expression of CD80 was still significantly reduced after subsequent CD40 ligation (*E. coli* 76.7% ± 1.5% vs. *B. vulgatus* 37.1% ± 0.6%; LPS^hi^ 79.0% ± 1.9% vs. LPS^lo^ 53.4% ± 3.7%) (Figure 2A). Analysis of the expression of CD86 on immature, semimature and mature DC upon CD40 ligation revealed a slight increase of CD86 expression in immature (9.1% ± 0.5% to 27.2% ± 4.7%) and semimature DC (*B. vulgatus*: 20.4% ± 5.1% to 42.5% ± 8.6%; LPS^lo^ 44.3% ± 7.9% to 58.7% ± 9.5%). However, the expression levels of CD86 on semimature DC after CD40 ligation did not reach the expression levels of mature DC (*E. coli:* 54.8% ± 6.7% to 59.9% ± 6.5%; LPS^hi^ 56.6% ± 3.3% to 61.3% ± 5.5%) (Figure 2B).

The MHC class II expression on immature, semimature and mature DC was slightly increased upon CD40 ligation. These changes, however, proved not to be statistically significant (data not shown).

As anti-CD40 antibodies used for ligation assays and anti-CD40 antibodies used for FACS analysis might compete for binding of CD40 we used the J558L/CD40L cell line to analyze the influence of CD40 ligation on the expression of CD40 itself on DC. In *B. vulgatus* treated semimature DC we found an increase of CD40 expression upon CD40 ligation (*B. vulgatus* 26.8% ± 7.4% to 55.0% ± 0.1%). In LPS^lo^ treated semimature DC we observed a similar effect, however, the increase in CD40 expression was statistically not significant (57.8% ± 6.6% to 63.5% ± 8.9%). Interestingly, CD40 ligation of semimature DC did not lead to an increase of CD40 resulting in as high expression levels as on mature DC (*E. coli* 77.1% ± 4.2%; LPS^hi^ 75.2% ± 4.0%) (Figure 2C).

### In DC semimaturation CD40L induced p38 phosphorylation is inhibited

**Figure 3:**
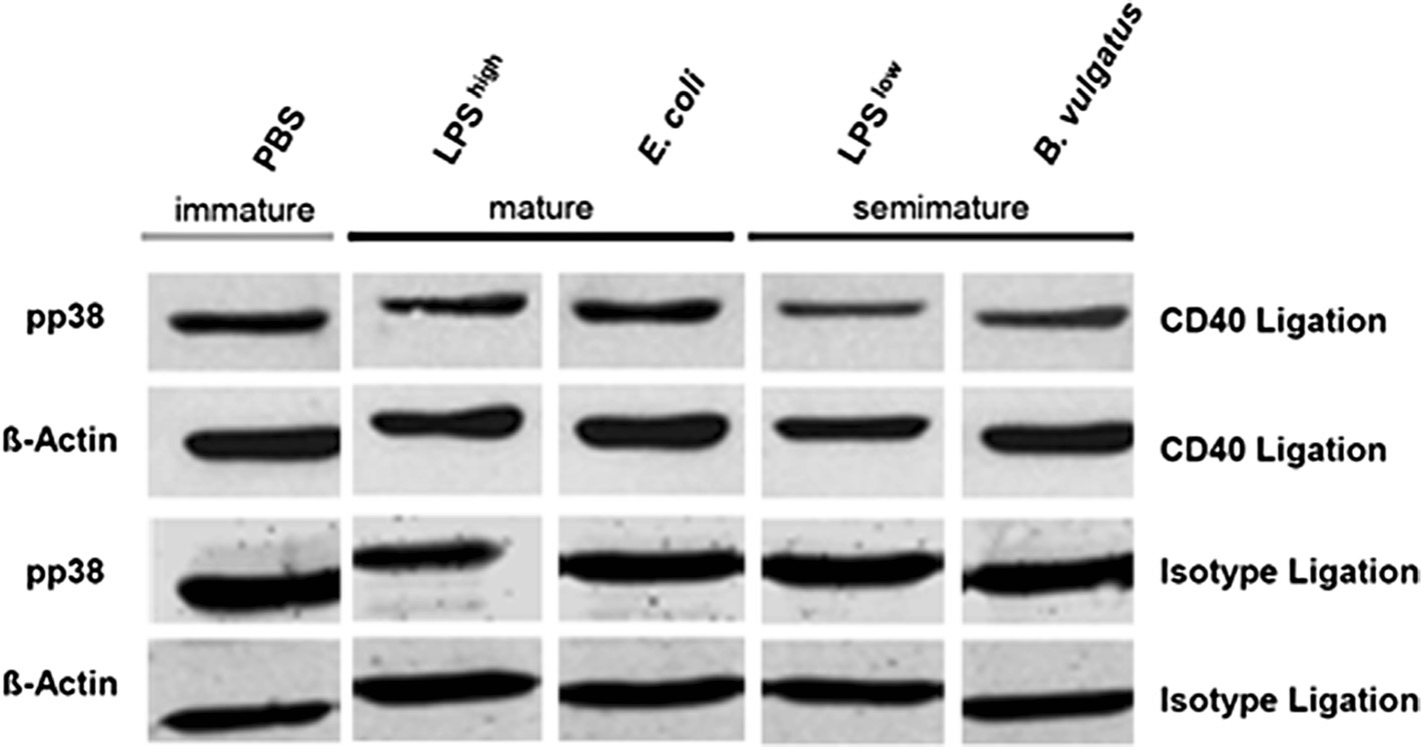
**Phosphorylation of p38 MAPK upon secondary CD40 ligation in immature, semimature and mature DC.** Wildtype BMDC were stimulated with B. vulgatus mpk or LPS 1 ng/ml (LPS^low^) to generate semimature DC and E. coli mpk or LPS 1 μg/ml (LPS^high^), respectively, to generate mature DC. Immature DC were maintained by incubation in the absence of further stimuli (PBS). After 24 hours, DC were re-stimulated with anti-CD40 mAb (1 μg/ml) for 15 minutes or treated with the isotype control (Isotype). The expression of pp38 was analyzed by Western blotting. As loading control, β-actin expression was determined. The results shown are representative of three independent experiments.

To analyze phosphorylation of the MAP kinase p38 in response to CD40 ligation, immature, semimature and mature DC were activated by CD40 ligation and pp38 levels were determined by Western blotting. CD40 ligation of immature and mature DC resulted in phosphorylation of p38, whereas in semimature DC p38 phosphorylation upon CD40 ligation slightly reduced (Figure 3).

**Figure 4:**
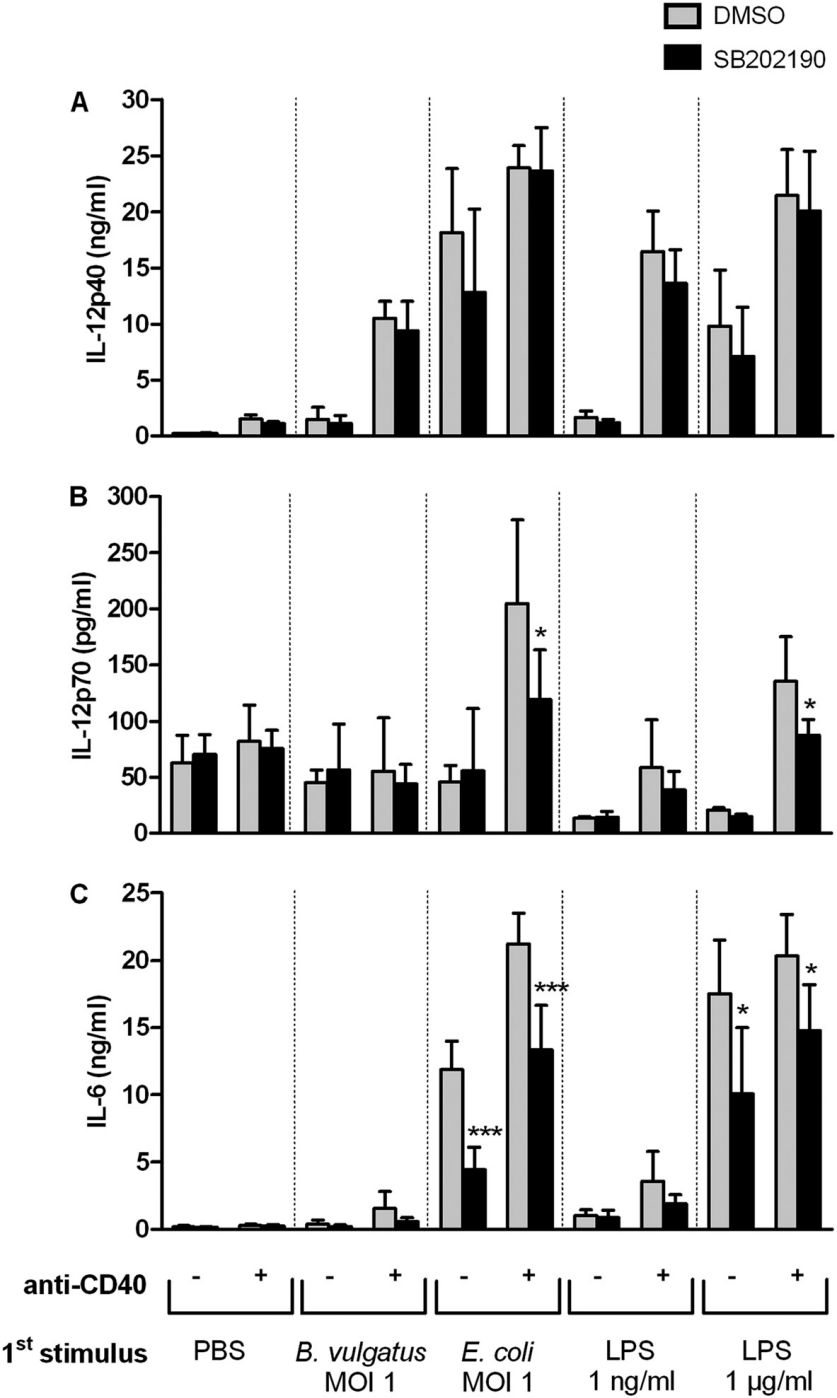
**Role of p38 MAPK on CD40L induced cytokine secretion in DC of different maturation status.** Wildtype BMDC were primed with B. vulgatus mpk or LPS 1 ng/ml to obtain semimature DC and E. coli mpk or LPS 1 μg/ml, respectively, to obtain mature DC. Immature DC were maintained by incubation in the absence of further stimuli (PBS). Subsequently, the DC were washed and treated with the p38 inhibitor SB202190 (2 μmol/l, black bars) or as a control with DMSO (grey bars) for 30 minutes, washed and re-stimulated with 1 μg/ml anti-CD40 mAb (+) or the isotype control (−). After 24 hours, the concentrations of IL-12p40 (**A**), IL-12p70 (**B**) and IL-6 (**C**) were measured in the cell culture supernatants by ELISA. The bars represent the mean values of three independent experiments, each performed as duplicate, ± SD. * p < 0.05, *** p < 0.001 for SB202190 compared to DMSO.

To investigate the biological relevance of p38 MAP kinase activation we treated immature, semimature and mature DC with the p38 inhibitor SB202190 prior to CD40 ligation. Levels of IL-12p40, IL-12p70 and IL-6 were determined in cell culture supernatants by ELISA (Figure 4 A-C).

Inhibition of p38 had no influence on the CD40L induced secretion of IL-12p40 by DC, independent of the maturation state (Figure 4A). However, in mature DC the production of IL-12p70 upon CD40 ligation was inhibited partially by SB202190. Inhibition of p38 did not influence the IL-12p70 expression pattern in immature or semimature DC as CD40 ligation did not induce any IL-12p70 secretion in these cells (Figure 4B). In mature DC, both spontaneous and CD40L induced IL-6 secretion levels were partially reduced by inhibition of the p38 MAP kinase. In contrast, IL-6 production of immature as well as semimature DC upon CD40 ligation was not significantly affected by inhibition of p38 (Figure 4C).

### In DC semimaturation ERK suppresses CD40L induced IL-12p40 production

**Figure 5:**
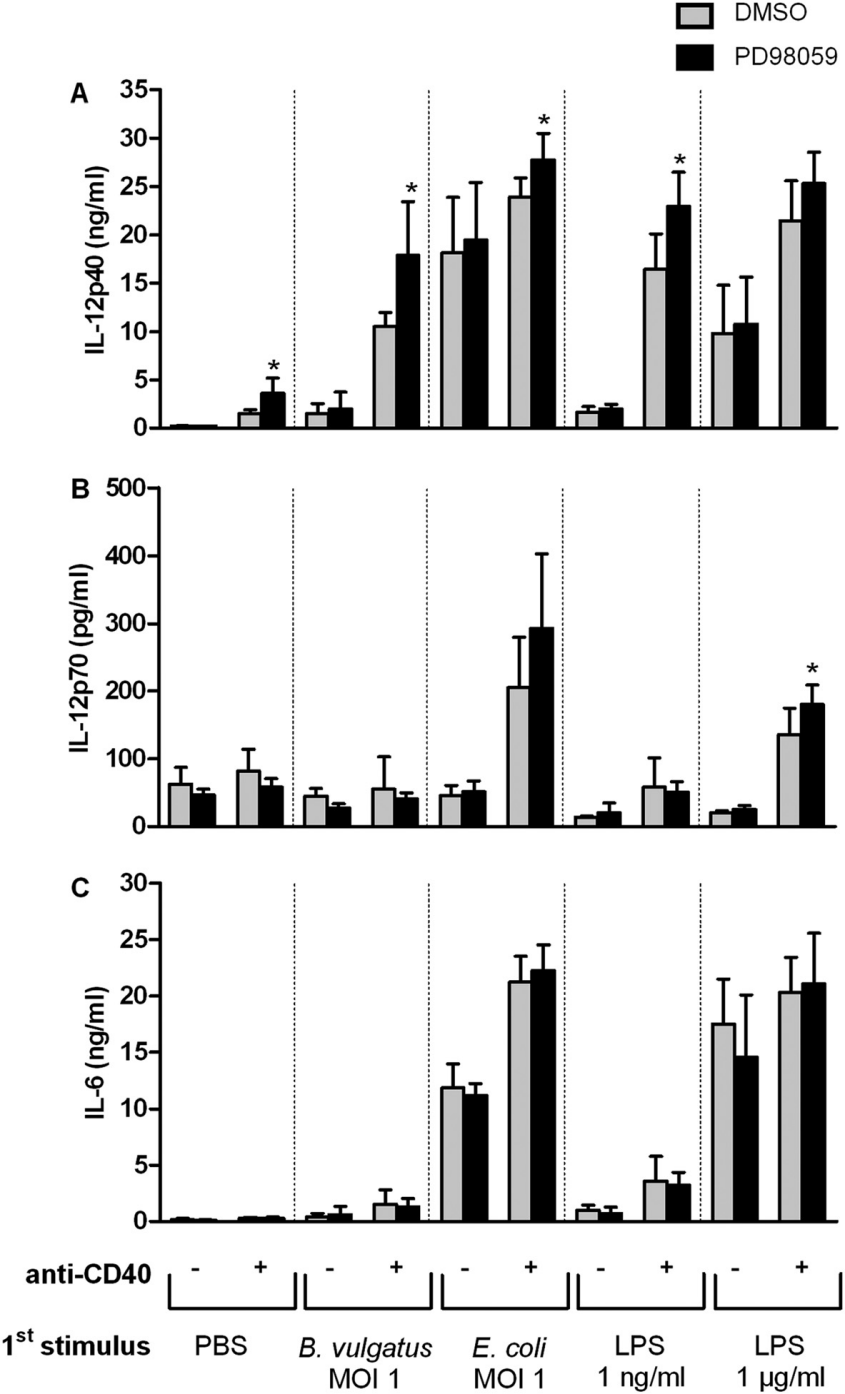
**Role of ERK on CD40L induced cytokine secretion in DC of different maturation status.** Wildtype BMDC were primed with B. vulgatus mpk or LPS 1 ng/ml to obtain semimature DC and E. coli mpk or LPS 1 μg/ml, respectively, to obtain mature DC. Immature DC were maintained by incubation in the absence of further stimuli (PBS). Subsequently, the DC were washed and treated with the ERK inhibitor PD98059 (50 μmol/l, black bars) or as a control with DMSO (grey bars) for 30 minutes, washed and re-stimulated with 1 μg/ml anti-CD40 mAb (+) or the isotype control (−). After 24 hours, the concentrations of IL-12p40 (**A**), IL-12p70 (**B**) and IL-6 (**C**) were measured in the cell culture supernatants by ELISA. The bars represent the mean values of three independent experiments, each performed as duplicate, ± SD. * p < 0.05 for PD98059 compared to DMSO.

To analyze the role of the extracellular signal regulated kinase (ERK) we used the ERK inhibitor PD98059. Upon CD40 ligation the inhibition of ERK resulted in a significant increase of IL-12p40 in immature and semimature DC but only a slight increase in mature DC (Figure 5A). In contrast, inhibition of ERK had not the ability to induce IL-12p70 production in immature and semimature DC and resulted in only slightly enhanced IL-12p70 secretion levels in mature DC (Figure 5B). The CD40L induced IL-6 production by DC was not affected by ERK inhibition, independent of the maturation state (Figure 5C). In line with this, analysis of pERK levels upon CD40 ligation of immature, semimature and mature DC showed similar levels independent of the DC maturation state (data not shown).

Taken together, our data showed that the semimature differentiation state of DC, induced by stimulation with *B. vulgatus* or LPS^lo^ can not be overcome by CD40 ligation.

## Discussion

In order to clarify the impact of CD40 expression on the T-cell activation capacity of semimature DC, we examined the effect of CD40 ligation on immature, semimature and mature DC. Semimature DC were induced by either stimulation with *B. vulgatus* or LPS at low concentration (1 ng/ml), and are characterized by a low positive expression of costimulatory molecules like e.g. CD40, secretion of only IL-6, and nonresponsiveness toward subsequent TLR activation [4,5].

In brief, we showed that CD40 ligation does not overcome DC semimaturation in terms of expression of activation surface markers and results in production of only IL-6 and IL-12p40, but not the bioactive form IL-12p70. The slightly reduced p38 phosphorylation levels in semimature DC as compared to mature DC might at least partially contribute to this effect. The expression of IL-12p40 turned out to be limited by pERK.

In line with other studies [24,25], we observed that on mature DCs no significant further increase in the expression levels of the already highly expressed costimulatory molecules CD40, CD80 and CD86 could be triggered upon additional stimulation by CD40 ligation.

Upon CD40 ligation immature and semimature DC expressed intermediate levels of CD40 CD80 and CD86, but did not reach the expression level of mature DC. However, the intermediate expression of costimulatory molecules was not associated with production of pro-inflammatory cytokines like IL-12p70.

It is known that immature DCs characterized by low expression levels of costimulatory molecules and lacking secretion of proinflammatory cytokines induce tolerance by promoting T-cell anergy, apoptosis or differentiation into T_reg_ cells via antigen presentation in the absence of costimulatory signals [26-29]. Additionally, CD40 deficient DCs or DCs with a suppressed CD40 expression were shown to have a reduced potential to activate T-cell proliferation and polarization in Th1 or Th2 direction [30-34]. This effect might also contribute to the inhibited T-cell activation induced by the intermediate expression of costimulatory molecules on semimature *lamina propria* (lp) DC of *B. vulgatus* monocolonized *IL-2*^*−/−*^ mice [3]. On the other hand it was shown that a high positive expression of costimulatory molecules in absence of pro-inflammatory mediators like e.g. TNF-α or IL-12p70 favours T-cell tolerance and suppression of T-cell activation. This type of DC is mainly induced by autocrine or paracrine stimulation with inflammatory mediators like e. g. TNF-α [35-38].

The cytokine secretion pattern upon CD40 ligation differed between immature/semimature DC and mature DC. In immature and semimature DC, CD40 ligation did not result in induction of IL-12p70 secretion, in contrast to mature DC where CD40 ligation led to increased IL-12p70 secretion. This is in line with other studies showing that TLR4 stimulation and CD40 ligation synergize in inducing IL-12 p70 secretion [25,39]. The additive microbial priming signals are necessary to trigger the production of the IL-12p35 subunit [40] which was shown to be not induced by exclusive CD40 ligation [41,42]. Additionally, these accessory stimuli have the potential to augment the CD40 expression on antigen presenting cells (APC) [43-45] which results in a more effective CD40 ligation. However, DC primed with *Bacteroides vulgatus* as a microbial stimulus do not secrete IL-12p70 upon CD40 ligation. This might be one mechanism accounting for the tolerogenic effects of *B. vulgatus* in maintenance of intestinal homeostasis [2,3]. As *Porphyromonas gingivalis* which is phylogenetically closely related to *B. vulgatus* signals mainly vial TLR2 [46], this might be also the main receptor for recognition of *B. vulgatus*. In turn, TLR2 activation is reported to result in transcription of the p40 but not the p35 subunit of IL-12p70 [1,47]. This might account for the induction of IL-12p40 but not p70 upon stimulation of *B. vulgatus* primed DC via CD40 ligation. The production of IL-12p40 in the absence of the p35 unit might result in the formation of IL-12p40 homodimers which are known to act as potent antagonists at the IL-12p70 receptor [48-50]. Additionally, in IL-12p40 transgenic mice Th1 responses are significantly reduced suggesting that also *in vivo* p40 functions as an IL-12 antagonist [51].

Upon CD40 ligation semimature DC produced significantly enhanced levels of IL-6 but not TNF-α (data not shown) or IL-12p70. This is in line with our previous studies showing a crucial role for IL-6 in induction of DC semimaturation and tolerance [4,5,52]. This is interesting as the secretion of IL-6 upon CD40 ligation by semimature DC might help to sustain the semimature differentiation state and influence the T-cell activation pattern. IL-6 plays an important role in T-cell differentiation through two independent molecular mechanisms. First, IL-6 stimulation of T-cells leads to an upregulation of nuclear factor of activated T cells (NFAT) [53], a transcription factor regulating IL-4 transcription [54] resulting in IL-4 expression, and thereby promotion of Th2 polarized T cell differentiation [55]. Second, IL-6 upregulates the expression of silencer of cytokine signaling (SOCS) 1 in CD4^+^ cells which inhibits IFN-γ signaling and thus Th1 differentiation [56]. The presence of IL-6 may shift the Th1/Th2 balance towards Th2 [55].

CD40 ligation of DC is known to result in phosphorylation of MAP kinases like e.g. p38 and ERK [57,58] and the ratio between pp38 and pERK is thought to play a crucial role in directing the cytokine secretion pattern of DC towards pro- or anti-inflammatory host responses [59-61]. CD40 ligation of mature DC resulted in phosphorylation of p38, inhibition of pp38 using the inhibitor SB202190 partially reduced of IL-12p70 and IL-6 but not IL-12p40 levels. Therefore, in mature DC pp38 might contribute to positive regulation of the p35 subunit of IL-12p70 [62]. This is in line with others showing that pp38 is important for production of IL-12p70 [61,63]. Additionally, pp38 is known to increase the stability of IL-6, TNF-α and IL-8 mRNA [22,23,64] which might result in increased secretion of these cytokines. Furthermore, via the mitogen and stress activated protein kinase (MSK) 1 pp38 is involved in NFκB activation [65,66]. In contrast, CD40L induced IL-12p40 secretion from mature DC has been shown to be independent of p38 phosphorylation, but dependent on the NFκB inducing kinase (NIK) [67].

As we observed an only slight reduction of p38 phosphorylation in semimature DC we hypothesize that inhibition of p38 phosphorylation due to DC semimaturation is only one of many factors that may affect in interaction with others the cytokine secretion pattern of semimature dendritic cells in response to secondary CD40 stimulation and thus their reduced pro-inflammatory capability [3-5]. The slight differences in the MAP kinase phosphorylation pattern in response to CD40 ligation might be based on differences in CD40 expression of immature, semimature or mature DC. A strong CD40 signal is known to preferentially activate p38, whereas weak CD40 signals are thought to favour ERK phosphorylation [60].

Inhibition of pERK during CD40 ligation turned out to have no significant effect of cytokine secretion in mature DC. In contrast, in semimature DC phosphorylation of ERK was at least partially responsible for limiting IL-12p40 expression. This is in line with others showing similar effects [68]. However, the Western blot analysis did not reveal significant differences of pERK levels in immature, semimature or mature DC. We speculate that in semimature DC the ERK activation might probably control the IL-12p40 production and therefore contribute to the limitation of the IL-12 p70 production. We are aware that this is highly speculative and that further work has to elucidate the role of ERK in DC semimaturation.

## Conclusion

We hypothesize that the inability of CD40 ligation in overcoming DC semimaturation might contribute to the tolerogenic phenotype of semimature DC and at least partially account for maintenance of intestinal homeostasis.

## Competing interests

The authors declare that they have no competing interests.

## Authors' contributions

AMG: Performance of experiments, analysis of data, writing of manuscript, AS: Performance of experiments, analysis of data, LK: Performance of experiments, AW: Performance of experiments, KG: Performance of experiments, JG: Performance of experiments, IBA: Designing of experiment, JSF: Designing of experiment, analysis of data, data interpretation, writing of manuscript. All authors read and approved the final manuscript.
